# Antiaging synergistic effect in noninvasive transdermal delivery of peptide loaded liposomes by low energy/frequency radiofrequency

**DOI:** 10.1016/j.ijpx.2024.100289

**Published:** 2024-10-05

**Authors:** Nanxi Xiang, Zeting Huang, Chunqiao Zhang, Jiahong Huang, Zhenyuan Wang, Jichuan Zhang, Chengyu Wu, Weihua Peng, Jiaheng Zhang

**Affiliations:** a Chongqing Innovation Center, Beijing Institute of Technology, Chongqing 401120, PR China; b Guangzhou Zhongzhuang Meiye Cosmetics Co., Ltd, Zhuhai 440105, PR China; c Sauvage Laboratory for Smart Materials, Harbin Institute of Technology, Shenzhen 518000, PR China; d College of Pharmacy, Shenzhen Technology University, Shenzhen 518118, China

**Keywords:** Antiaging, Low energy/frequency radiofrequency, Liposomes, Peptide, Tea extract

## Abstract

Low energy/frequency radiofrequency (LRF) combined with the transdermal delivery of liposome (L) encapsulated antiaging peptides technology is a remarkable, newly developed physical noninvasive transdermal penetration technique; it is considered a highly efficient, comprehensive and safe technology. In this study, our objective was to evaluate the physical and chemical mechanisms underlying the efficacy of this innovative technique involving a combination of LRF and L, termed LLRF, that exerts a synergistic anti-aging effect on human skin, via an animal experiment. Physical and chemical analyses indicated that a relatively stable liposome with a uniform nano-size, which was formed, possessed good transdermal permeability that was 2.74 folds higher than that of the free peptide (F). LLRF exhibited a higher transdermal permeation performance that was of 3.65 folds higher than that of the free one, which was substantiated via confocal laser scanning fluorescence microscopy. The mouse UVB photoaging model trial confirmed that the LLRF technology exerted a significant synergistic effect compared to liposome technology, or free peptide, by downregulating inflammatory factors (IL-6, TNF-α), inhibiting the mRNA and protein expression of matrix metalloproteinases (MMP1, MMP3), promoting the mRNA and protein expression of related collagens (Procollagen, Col1α1 and Col3α1), and repairing the stratum corneum barrier function, as evidenced by trans-epidermal water loss (TEWL), skin cuticle hydration (SCH), and decreased expression of β-gal, an aging marker. These findings indicated that photoaging skin can be effectively and comprehensively rejuvenated, and that even photodamage can be reversed, thereby restoring the original physiological characteristics of healthy skin. Clinical tests have confirmed that although liposome technology is an effective antiaging method which helps exert tightening and anti-wrinkle effects on human skin, LLRF is an even more effective anti-aging technique. This study reveals a highly effective technique involving a combination physical and chemical therapy that may be utilized for antiaging purposes as well as repairing lightly damaged skin, and can be made readily available in the future.

## Graphical abstract

**Figure:**
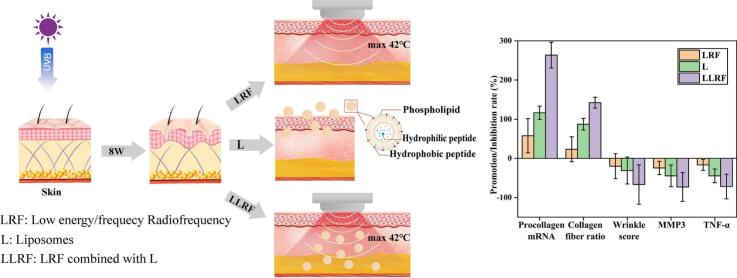

## 1 Introduction

In humans as well as other biological organisms, aging is an inevitable process and a natural phenomenon. However, various methods have been developed in an attempt at delaying or inhibiting the acceleration of aging under natural as well as harsh environmental conditions. Altering the characteristics of aging from the perspective of skin aesthetics is sought after by a majority of people, and this may be achieved via one of two therapeutic methodologies: surgical methods; or non-surgical methods, such as that via a transdermal delivery system (TDS). TDS is one of the most popular non-surgical methods, on account of low drug toxicity, less administration frequency, better drug compliance, avoidance of liver first-pass effects, and the absence of pain, such as that caused during the administering of hypodermic injections (Amjadi et al., 2018). Ensuring the transdermal penetration of common drugs or their pharmaceutical preparations involves overcoming the stratum corneum barrier effect of the skin, which poses a major issue. The difference between the hydrophobicity and hydrophilicity of the dermis and the epidermis, respectively, makes guaranteeing the efficacy of drugs or their activity, stability and long-term release extremely challenging. Thus, solving the transdermal delivery of water-soluble or active macromolecular drugs poses a major challenge for researchers. Transdermal delivery strategies include niosomes, self-assembly techniques (Emad Eldeeb et al., 2019), nanostructured lipid carriers (Kapoor et al., 2019), nano-emulsion techniques (Alkilani et al., 2018), iontophoresis techniques (Ita, 2016a), electroporation techniques (Ita, 2016b), microneedles techniques (Sabri et al., 2019; Zu et al., 2017), and ultrasound techniques (Manikkath et al., 2017; Ita and Popova, 2016), among others. These transdermal penetration technologies are associated with both advantages and limitations, such skin barrier damage caused by microneedles, skin irritation due to ionic electro-osmosis, cytotoxicity and reagent-related irritation caused by chemical enhancers, and iontophoresis, due to the use of ionized compounds.

Radiofrequency (RF) is a powerful and multifunction technique based on thermos-biological effects. The main mechanism underlying RF involves heating the dermis while preserving the epidermis (Bonjorno et al., 2020). Due to being associated with only a few side effects and good drug compliance, RF is widely used, either alone or in combination with other techniques, in medical and medical aesthetics fields. Based on differences between the blood supply to cancer tissues and normal tissues, RF is used to selectively thermally ablate tumor tissues (LeVeen et al., 1980). RF has been used to treat acne scarring and hyperhidrosis (Weiner, 2019) while low temperature RF has been applied to treat tracheobronchial amyloidosis (Kosnik et al., 2023). In addition, RF is applied to relieve pain, muscle spasms, inflammatory reactions and welling states (Goats, 1989). Combined use of RF and proton pump inhibitors reportedly yielded results that were superior to other therapies aimed at gastroesophageal reflux disease (Dugalic et al., 2020). RF catheter ablation was developed during the course of adopting cardiac arrhythmia therapies (Hindricks, 1993). RF may be noninvasively applied to rejuvenate facial and neck skin for aesthetic purposes (Sadick, 2016). RF has been applied to achieve weight loss, abdominal circumference and body weight (Fritz and Salavastru, 2017). Furthermore, thermo-chemotherapy may possibly be achieved by exposing nano-encapsulation drugs to suitable hyperthermia energy (Fritz and Salavastru, 2017). Considering that the outcome of applying a single technique is always limited to an effect specific to that technique, the combined application of two or more techniques may be a more promising prospect, on account of synergistic effects that may possibly occur (Beik et al., 2016).

However, RF techniques are usually used at high frequency/energy (Thermage 6.78 MHz, Slimager 40.68 MHz), where the use of such high energy RF frequently leads to side effects, such as mild to moderate edema and erythema, although these are generally of a transient nature (Hasan et al., 2020). Furthermore, the special designs as well as high prices that are involved prevent high frequency/energy RF from being widely used. Although it is safer and readily available, compared to high frequency/energy RF, low frequency/energy RF rarely receives attention.

Polypeptide are secondary structures formed by amino acids linked via peptide bonds. They are used in a wide range of physiological, medical and skin care applications. Acetyl hexapeptide-8 (AH-8) and palmitoyl pentapeptide-4 (PP-4) are peptides that exert good anti-aging effects. However, due to the large molecular weight and hydrophilic characteristics of such peptides, it is difficult to administer them directly via traditional transdermal methods. In this study, we developed a new technique ((LLRF) for applying TDS in anti-aging therapy, by combining low energy/frequency radiofrequency (LRF) with transdermal delivery of liposome encapsulated peptides. The new LLRF technique is simple, easy to operate, safe, and effective, and may thus be easily popularized. In this study, our main purpose was to explore whether household type LRF (0.3 MHz, 12w, tripolar RF) facilitates the transdermal delivery of peptides, and whether it exerts a synergistic effect on anti-aging in an animal photoaging model, thereby indicating that LLRF, which combines LRF with liposome TDS technology, shows potential as a new technique in anti-aging therapy.

## 2 Materials and methods

### 2.1 Materials

Soybean phospholipid (Tywei Pharmaceutical Co., Ltd., Shanghai, China); cholesterol (Fengtai Biotechnology Co., LTD, Shandong, China); 1,2-pentanediol (Adamas, Switzerland); ethoxy diglycol (Dow, US); 1,3-propylene glycol (Dupont, US); PEG-40 hydrogenated castor oil (BASF, Germany); palmitoyl pentapeptide-4 (PP-4), acetylhexapeptide-8 (AH-8) and palmitoyl tripeptide-1 (Ruidelin Biotechnology Co., Ltd., Shenzhen, China); FITC-acetyl hexapeptide-8 (FITC-AH-8) offered by Xi'an qiyue biology technology CO. Ltd.; tea extract water (Shinehigh Innovation technology CO., Ltd., Shenzhen, China).

### 2.2 Liposome preparation

Phase A: soybean phospholipid, cholesterol, 1,2-pentanediol and PEG-40 hydrogenated castor oil were mixed together and dissolved by heating to 50 °C. Phase B: palmitoyl pentapeptide-4, palmitoyl tripeptide-1 was dissolved in ethoxy diglycol at 50 °C. Phase C: AH-8, 1,3-propylene glycol and tea extract water were mixed together at room temperature, and heated in a water-bath at 50 °C. Phase C was added slowly to the mixture of phase A and B at 50 °C, and homogenized 4 times in a high pressure homogenizer (Antos Nano technology CO., Ltd., Suzhou, China) at a pressure of 500–700 bar at room temperature. Finally, the above liposomes were filtrated using a 0.45 μm microporous filter membrane.

### 2.3 Characterization of physicochemical parameters

The hydrodynamic diameter and zeta potential of liposome peptides were measured using a Litesizer 500 (Anto Paar, Switzerland), while the structural features were confirmed via transmission electron microscopy (TEM, HT7700, Hitachi, Japan). Encapsulation efficiency (EE) was determined using high performance liquid chromatography (HPLC, Agilent 1220 Infinity II, USA), and AH-8 and PP-4 contents were measured by follows: a 2 g liposome samples were loaded into an ultrafiltration tube (Amicon Ultra Centrifugal Filter Units, MWCO 10,000, Merck Millipore) and centrifuged at 4 °C, 2740 *g* for 60 min (TGL-23, Sichuan Shuke Instrument Co., LTD, China), then the centrifugal liquid (unencapsulated peptide) was detected by HPLC, on the other hand, a same quantity of liposomes were deal with Triton X-100 (a final concentraion of 1 %), then ultrasonic treatment for 10 min, and the total peptide content was detected by HPLC. Gradient elution procedures were performed with the following mobile phases: methanol: 0.1 % Trifluoroacetic acid (TFA) water = 10:90 (0 min); methanol: 0.1 % TFA water = 10:90 (2.5 min); methanol: 0.1 % TFA water = 80:20 (17.5 min); methanol: 0.1 % TFA water = 80:20 (22.5 min); methanol: 0.1 % TFA water = 10:90 (22.6 min); methanol: 0.1 % TFA water = 10:90 (25.0 min). An Inertsil ODS chromatographic column sized, 4.6 mm × 250 mm × 5 μm, UV detection at 210 nm, an injection volume of 10 μL, a flow rate of 1.0 mL/min and a constant column temperature of 35 °C were adopted for this procedure.

### 2.4 Transdermal absorption test

Qualitative transdermal absorption was characterized via the amount of AH-8, while the quantitative transdermal penetrate was characterized via FITC-AH-8. In vitro porcine skin of pig ear-back was used to conduct transdermal drug administration due to its close similarity to human skin. Skin was incubated at different time points for 4 and 8 h, according to the Franz diffusion method, and collected at 4 and 8 h, respectively. Next, the pig skin was cut into particles with a diameter of less than 0.2 cm, incubated in a methanol-water solution (methanol: water = 1:1(*v*/v)) for 4 h, subjected to homogenizer treatment at 4 °C as well as sonication for 10 min at room temperature, and repeat extracted twice, using a 0.22 μm membrane filter. The amount of active ingredients was detected via high performance liquid chromatography (HPLC) as mentioned above. Laser scanning confocal microscopy (LSCM, BioTek Lionheart FX, Agilent, USA) was used for the quantitative analysis of transdermal penetration ability, and AH-8 in liposome was replaced by FITC-AH-8 in this situation for fluorescence observation.

### 2.5 Fourier transform infrared spectroscopy (FTIR)

Based on methods described by Samir Mitragotri (Lolis and Goldberg, 2012), FTIR (FTIR-850, Tianjin Gangdong Sci.& Tech. Development Co., LTD, China) measurement was performed on the stratum corneum (SC) of porcine skin, in follows conditions: LRF for 10 min, incubation in diethylene glycol monoethyl ether (DGME)(20 %, w)/AH-8(0.1 %, w) solutions for 24 h, LRF treated 10 min after incubation in AH-8 liposomes (0.1 %, w) for 24 h, directly incubated in AH-8 (0.1 %, w) liposomes for 24 h, incubated in AH-8 solutions (0.1 %, w) for 24 h, incubated in PBS solutions for 24 h, respectively. Briefly, the skin was cut into 3.0 cm × 3.0 cm section, defrosted at room temperature, and subjected to a 24 h transdermal treatment at 32 °C ± 1 °C or 10 min of LRF treatment, and washed with PBS solution heated in a water bath for 3 min at 65 °C ± 2 °C, following which the epidermis was separated from full skin using tweezers. The stratum corneum of the skin was digested with trypsin (0.25 %), washed with PBS, dried at room temperature for 72 h, and cut into 1.5 cm × 1.5 cm slices, following which FTIR analysis of SC samples was performed.

### 2.6 Antiaging experiment

#### 2.6.1 Model construction and grouping

Six-week-old male Balb/c nude mice weighing 18–20 g were purchased from Nanjing Junke Bioengineering Co., LTD. All mice were kept in an SPF environment, given standard diet and water, and exposed to a 12 h light/dark environment cycle, at a temperature of 25 ± 2 °C, and a relative humidity 0f 50–60 %. Ultraviolet light was applied to the back skin of mice every other day for 8 weeks to mold a skin phatoaging in mice, as described by Shiqi Hu (Karande et al., 2005). The total dose of UVB exposure was approximately 80 MED for 8 weeks.

Experimental grouping was as follows (mice: Balb/c nude mice, *n* = 6 mice per group):

(1) Negative control group: no interference, marker as ‘NC’; (2) UVB aging modeling group: normal saline coating, marker as ‘M'; (3) UVB aging model + low energy/frequency radio frequency (RF) treatment group: coated with normal saline and treated with a radiofrequency instrument for 5 min, labeled as ‘LRF’; (4) UVB aging model + liposome containing peptide reagent treatment group: the back skin of mice was coated with the reagent (contain 0.01 % AH-8, 0.002 % PP-4) and labeled as ‘L'; (5) UVB aging model + liposomes + LRF treatment group: the back skin of mice was coated with peptide reagent, treated with a radio frequency instrument for 5 min and labeled as ‘LLRF; and (6) UVB aging model + VC treatment group (positive control): the back skin of mice was coated with Retinoic acid (0.1%) and labeled as ‘PC’.

Note: following formation of the mold, 6 mice were randomly selected for a pre-experiment and treated thrice with a triple dose of LRF treatment, and monitored to identify any significant harmful changes within a week, before starting the experiment proper. The frequency of treatment adopted was twice a week, where the above treatments were used once every three days, with the treatment period lasting 4 weeks.

#### 2.6.2 Pharmacodynamic evaluation

Wrinkle scoring criteria, adopted from Inomata (Hu et al., 2019) was used to assess the restorative effect exerted on photoaged skin overall. Mouse skin wrinkles were graded as follows (Hu et al., 2019): No wrinkles, score 0; A few shallow wrinkles across the back skin observed occasionally, score 2; Shallow wrinkles across the whole surface of back skin, score 4; Some deep, long wrinkles across the back skin, score 6; Deep, long wrinkles across the whole surface of back skin, score 8.

#### 2.6.3 Histological analysis

HE staining (Inomata et al., 2003) was adopted to observe histopathological changes in the skin, in addition to which epidermal and dermal thicknesses were measured. The paraffin-embedded skin specomens were anlysis by Masson stain kit (Adamas life) for the investigation of the changes in skin collagen fibers (Inomata et al., 2003).

#### 2.6.4 Immunofluorescence for β-galactosidase (β-Gal) observation

The expression of β-Gal (a skin aging marker) in skin tissue was detected via immunofluorescence. Cy3-labeled IgG, was used as second antibody, DAPI was used to stain the nucleus, following which a fluoroscopic scan was performed at 340 nm (excitation wavelength) and 480 nm (emission wavelength) for DAPI and 510–560 nm (excitation wavelength) and 590 nm (emission wavelength) for Cy3.

Note: 6 mice were selected from each group for the above pathological analysis of sections, and 10 fields of view were selected for each mouse section that was scanned to measure epidermal and dermal thickness, and to analyze areas that were positive for collagen fiber and relative β-Gal expression.

#### 2.6.5 Skin barrier function examination

A cutometer, MPA-580, with a Tewameter TM300 probe and a Corneometer CM 825 probe was used to measure transepidermal water loss (TEWL) and cuticle hydration (SCH), after 4 weeks of therapy. Under a constant temperature of 25 °C and constant humidity of 50 %–60 %, fresh skin sections of mice were selected, and a 2 × 2 cm area was selected in the middle of the abdomen for the detemination of TEWL and SCH values. Each mouse was measured once, and the average value of 6 mice was taken as the result.

#### 2.6.6 Western blot test

Western blot analysis was performed to detect the protein expression levels of Pro-collagen, Col1α1, Col3α1, MMP-1, MMP-3, IL-6 and TNF-α. A 20 mg skin tissue was treated with RIPA lysate, and homogenized at 4 °C for 30 min, following which the supernatant was centrifuged at 12000 rpm for 10 min, and total tissue protein was extracted. The primary antibodies used were: Pro-collagen II (1:500), Col1α1 (1:1000), Col3α1 (1:1000), MMP-1 (1:1500), MMP-3 (1:800), IL-6 (1:1000), TNF-α (1:500) and GAPDH (1: 10000). Following incubation at 4 °C overnight, TBST rinsed 3 times, 10 min/ time. The next day, the preparation was incubated with Goat Anti-Rabbit IgG (H + L) HRP (1:15000) or Goat Anti-Mouse IgG (H + L) HRP (1: 10000), at room temperature for 1 h, and rinsed in TBST thrice, 10 min at a time. After chemiluminescence development on the ECL substrate, band integral absorbance value was quantitatively analyzed using Quantity One software, and the expression of protein was measured via the integral absorbance ratio.

#### 2.6.7 Real-time PCR

The mRNA levels of collagen II, Col1α1, Col3α1, MMP-1 and MMP-3 were detected using Real-time PCR. A 30 mg of skin tissue was immersed in 1 mL of non-frozen tissue RNA preservation solution and placed in a refrigerator at 4 °C overnight. Total tissue RNA was extracted using the TRIZOL method. A fluorescence quantitative PCR instrument was used for on-board amplification detection (two-step amplification was adopted due to the product being less than 300 bp), and the reaction conditions were as follows: ①Predenaturation: 95 °C/3 min; ②Denaturation: 95 °C/3 s, annealing/extension: 60 °C/30 s, and ③A total of 40 cycles; According to Ct values, 2^-△△Ct^ was used to represent mRNA relative levels. Sequeces of primers used in this study are listed in Table S1.

### 2.7 Clinical experiment

VISIA-CR and PRIMOS-CR were used to measure eye wrinkles: One week before the test, the subjects stopped using any cosmetics or topical drugs, and having washed their faces with water at home the night before the test, the subjects did not apply any skin care products on the morning of the test. The subjects who were found to be qualified after screening filled in the informed consent. Subjects cleaned their faces with a facial cleanser in the lab, and rested for 20 min at a temperature of 21 ± 1 °C and a relative humidity of 50 ± 10 %. The number, length, area and depth of wrinkles were recorded and test samples were issued with instructions for use. The facial data of the sample were re-measured after 14 and 28 d. A Skin elasticity tester, MPA 580, was used to measure the tightness of skin: the test process was the same as the one used for eye wrinkle measurement, the only difference being the detector.

### 2.8 Statistical analysis

All data were analyzed using the SPSS 19.0 software package. All data are expressed as mean ± standard deviation (SD). One- way ANOVA was used to compare significance of differences among all groups, except for skin wrinkle score data. Differences that were significant (*P* < 0.05), were analyzed for pair-wise comparisons between groups, using LSD (least significant difference), with P < 0.05 being considered statistically significant. The Kruskal-Wallis nonparametric test was used to compare skin wrinkle scores among all groups. Differences that were significant (P < 0.05) were analyzed for pairwise comparison between groups using the Mann-Whitney *U* test, with P < 0.05 being considered as statistically significant.

## 3 Results and discussion

### 3.1 Characterization

**Fig. 1:**
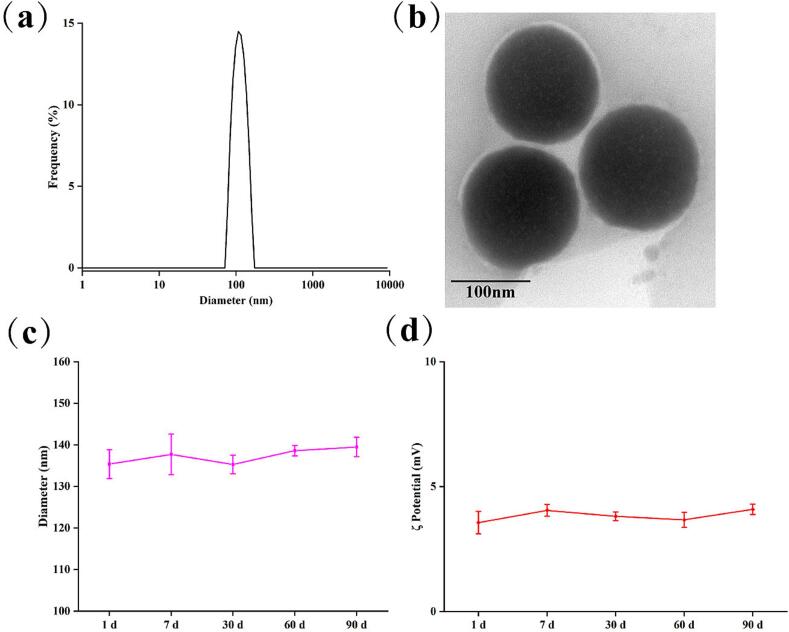
DLS measurement of liposomes size(a) and TEM images of liposomes, scale bar: 100 nm (b), and the physicochemical parameter stability of hydrodynamic diameter (c) and ζpotential (d) at room temperature.

The physicochemical parameter, pH, was determined using a Mettler Toledo FE28. The size, distribution and zeta potential of liposomes were measured via dynamic light scattering. Encapsulation efficiency (EE) was measured using the ultrafiltration tubes method and quantified via high performance liquid chromatography (Supporting Information). The morphology of liposomes was observed via transmission electron microscopy. The physicochemical parameter stability of peptide liposomes and TEM images were measured at room temperature (Fig. 1).

Measurement of the physicochemical parameters of peptide liposomes indicated that the prepared liposomes had a neutral pH (7.36 ± 0.02), and a hydrodynamic diameter of approximately 135.38 ± 3.46 nm with monodispersing (0.093 ± 0.005), with the originally formed liposome particles carrying a zeta potential of approximately 3.56 mV ± 0.45 mV (Table S2). The HPLC method was a suitable way for AH-8 and PP-4 quantitative analysis (Fig.S1), by which the EE was detected, and it shows that the encapsulation efficient of water-soluble antiaging active substances, such as AH-8 (EE_AH-8_), was lower (∼ 68 %), whereas the encapsulation efficient of hydrophobic antiaging drugs, such as palmitoyl pentapeptide-4 (EE_PP-4_) was higher (> 96 %) (Table S2), those findings were substantiated by those of Kim (Feng et al., 2022). The long-term observation test, lasting 90 d, indicated that the physicochemical parameters and EE values of prepared peptide liposomes remained stable at room temperature. Such high stability of peptide liposomes was closely associated with the formation of particles of smaller size and uniform size distribution, the smaller particle size being conducive to stability (Kim et al., 2019). The zeta potential of blank liposomes without peptides was −25.3 ± 2.11 mV, indicating a hydrophobic modified pentapeptide that was interspersed in the lipid bilayer membrane structure with the hydrophilic group directed out towards the water phase, which changed the membrane zeta potential of the liposomes. Although the absolute value of zeta potential usually indicates the stability of a particle system (Guner and Oztop, 2017), a system with a zeta potential of low absolute value is usually unstable due to week electrostatic repulsion. However, although the liposomes prepared via high pressure homogenization in this study exhibited a zeta potential of low absolute value, they remained very stable, which may be attributable to their smaller particle size and uniform dispersion. The hydrogen bonds among peptides or water provide a suitable level of viscosity that contribute to the stability of liposomes. Their regular spherical structure with a homogeneous size about 130 nm was once again confirmed via TEM imaging (Fig. 1(b)), which showed that the prepared peptide liposomes had a uniform size, which was consistent with the measurement results of DLS (Fig. 1(a)). It also indicated that the stability of liposomes was more closely related to particle size and distribution.

### 3.2 Quantitative of transdermal penetration

**Fig. 2:**
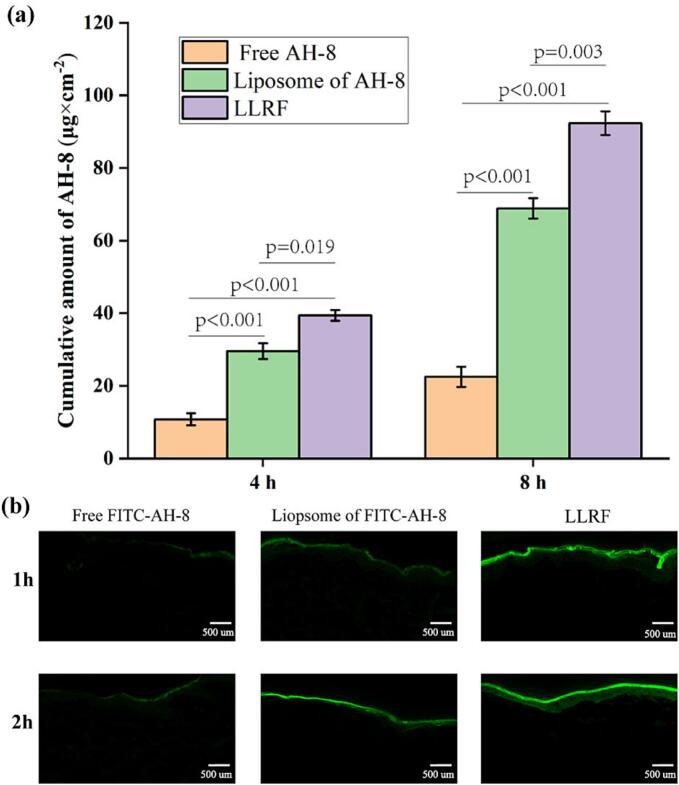
Cumulative amount of AH-8 in transdermal penetration test (a) and fluorescence photographs for observation of the pro-permeation effect of L and LLRF technique on FITC-AH-8 (b).

The transdermal retention content of AH-8 was detected via the Franz diffusion pool experiment in order to estimate its transdermal ability in different situations. The above mentioned HPLC method was used for quantitative analysis in this section. In 4 h, the amount of peptides retained in the liposome capsulated system was increased significantly by 274 % (2.74 times), compared with that of the non-encapsulated system. Meanwhile, the transdermal retention of peptide in the combined LRF machine and liposome technique was significantly increased by 365 % (3.65 times), compared with that of the non-encapsulated system (Fig. 2 (a)). Furthermore, there was a significant difference between the penetration abilities corresponding to the use of the LRF technique combined with peptide liposomes and that of peptide liposomes alone. These results indicated that although the effect exerted by the liposome system promoted a suitable level of permeability in the peptide (AH-8), the level of permeability promoted by the LLRF technique was outstanding. Skin tissue changes examined via HE staining (Fig. S2), indicated that there was no significant difference among the three transdermal methods before and after the treatment of pig skin tissues, all being in good shape and undamaged, indicating that the combined technology of LLRF was safer in this study.

A confocal laser scanning microscope (CLSM) was used to observe the pro-permeation effect of different transdermal methods. FITC-acetyl hexapeptide-8 (FITC-AH-8) offered by Xi'an qiyue biology technology CO. Ltd., was used to replace AH-8 for tracking penetration ability. The results of 1 and 2 h tests of transdermal penetration activity are shown (Fig. 2 (b).). The 1 h transdermal test indicated that free FITC-AH-8 showed shallow transdermal fluorescence intensity which was limited to the epidermal layer, whereas fluorescence intensity below the epidermal layer was observed in both L and LLRF systems. The results of 2 h fluorescence transdermal penetration showed that, a certain thickness of free FITC-AH-8 could be observed in the epidermis and sub-epidermis, while the fluorescence intensity in both the L and the LLRF systems showed some thickness under the dermis, even penetrating into hair follicle in LLRF, which indicated a significant improvement in 2 h compared with 1 h for free FITC-AH-8, although only a minor difference was observed between the LLFR and L systems. A previous study (Guner and Oztop, 2017) has revealed that the reason underlying this result was the absence of a linear relationship between the transdermal retention content of the active substance and time. Therefore, it is speculated that the first transdermal permeability process undergoes a period of relaxation, followed by a period of stable penetration and a period of permeation mitigation (retardation). The relaxation period reflects the time required for active ingredients to overcome the skin barrier, while stable penetration represents the formation of a stable state. The retardation period, in which a concentration gradient difference or a relatively stable osmotic pathway is formed, is the process wherein the concentration of the active ingredient is reduced leading to the retardation of diffusion. Within 1 h of this test process, the skin tested using the FITC-AH-8 solution remained in the early relaxation phase, with slow penetration, whereas the liposome which show good compatibility with the skin penetrated faster, indicating that the combined LRF instrument had promoted further penetration., All three transdermal methods achieved stable transdermal diffusion within 2 h, resulting in the relative fluorescence intensity being significantly improved, compared with that in 1 h. Thus, the results of fluorescence transdermal analysis showed that both the liposome encapsulation system and the RF combined with liposome system had exerted obvious permeability promoting effects.

### 3.3 FTIR analysis

**Fig. 3:**
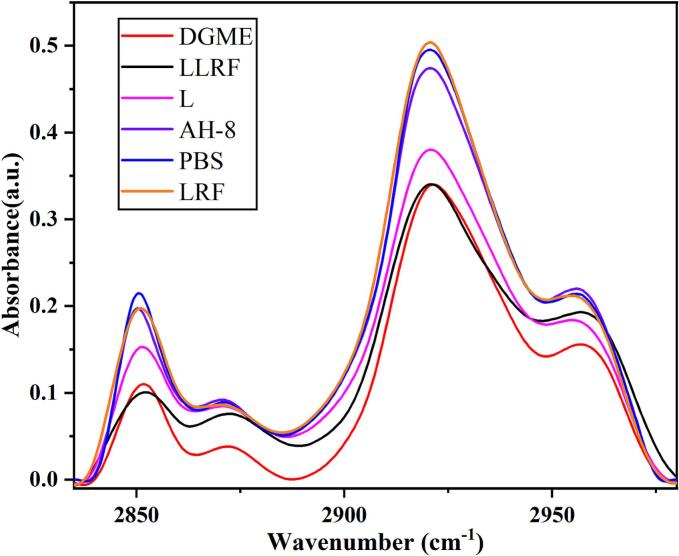
IR spectra of SC treated by diethylene glycol monoethyl ether (DGME)/AH-8 (red), combined use of low energy/frequency radiofrequency delivery of liposome encapsulated of peptides (LLRF)/AH-8 (black), liposome of AH-8 (L) (pink), AH-8 solutions (AH-8) (purple), PBS solutions of AH-8 (PBS) (blue), low energy/frequency radiofrequency delivery of free AH-8 (LRF) (yellow). (For interpretation of the references to colour in this figure legend, the reader is referred to the web version of this article.)

FTIR spectroscopy analyses were performed to gain insights into the different processing methods on stratum corneum and epidermal structures (Zhang et al., 2020). The characteristic absorption peaks at 2852 cm^−1^ and 2922 cm^−1^ in infrared reflected the symmetric and asymmetric stretching of CH_2_ in phospholipids and fatty acids of the SC. The reduction in peak intensity indicated paracellular transport via lipid extraction (Olsztyńska-Janus et al., 2018). The FTIR spectroscope results are shown in Fig. 3, the lowest absorption intensity corresponding to LLRF and DGME treated SC implied a strong lipid interaction, while L treated SC showed a moderate absorption intensity indicating a relatively week interaction, while the considerably equivalent intensities of SC treated with LRF, PBS, AH-8 solutions indicated that they had not exerted an interference effect on SC. This result revealed that although LRF had not exerted a significant impact on SC, it may have accelerated liposome interaction with SC. After LLRF treatment, there is no significant adverse effect on the skin, so it is speculated that the liposome penetration delivery by LLRF is a process that has no significant negative effect on the skin and can be recovered immediately after treatment. In noninvasive transdermal delivery of liposome by weak electric current-iontophoresis, involves two mechanisms, electromigration and electroosmosis (Hasan et al., 2020), both of two mechanism support the delivery of charged drugs especially, positively charged drugs, otherwise, the delivery of neutral molecules was suitable via passive diffusion following electroosmosis by weak electric current. In our study, no current as a driving force, radio-frequency thermal effect as the driving force instead. Radio-frequency thermal effect probably have a similar function to activate the intracellular signaling pathway of unique endocytosis in skin cell for liposome delivery. LLRF offers the driving force for migration rather than directly enhancing skin permeability. In contrast to traditional physical permeation enhancers, it is straightforward to operate and has no adverse impact on the SC of the skin. Hence, we are convinced that this permeation enhancement approach that does no harm to the skin barrier is safe and effective.

### 3.4 UVB mouse photoaging model

#### 3.4.1 Skin morphology observation

**Fig. 4:**
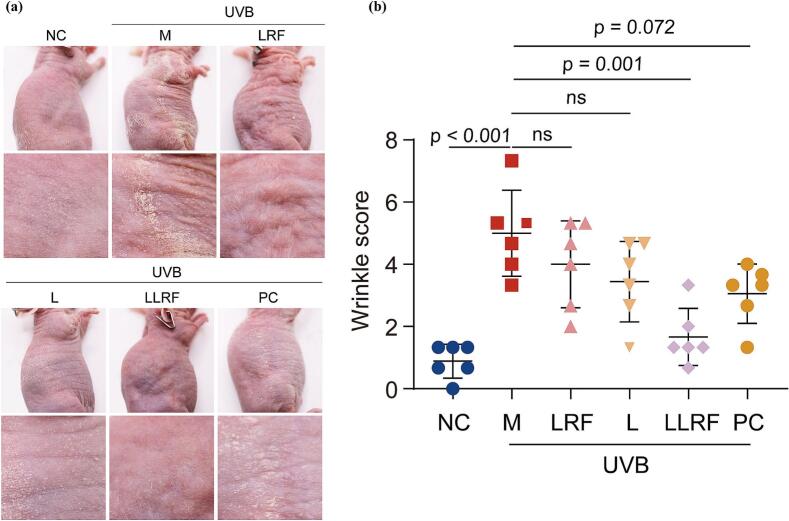
The morphology of mouse skin wrinkles(a) and the assessment of wrinkle scores (b) (*n* = 6). NC: negative control group; M: UVB aging modeling group; LRF: low energy/frequency radio frequency treatment group; L: liposomes of peptide reagent treatment group; LLRF: liposomes of peptide used before low energy/frequency radio frequency treatment group; PC: retinoic acid (0.1 %) treatment group. (the same as the follow).

Images of mouse wrinkle morphology were obtained via a HD camera, following which wrinkle scores were assessed. Few wrinkles were observed in the NC and LLRF groups, in contrast to significant pathological changes (wrinkles, hyperkeratosis and scabs) observed in the skin of hairless mice following a certain amount of UVB irradiation (Fig. 4), which made self-recovery within 4 weeks difficult; on the other hand, a mild therapeutic effect was evident in the L, LFR and retinoic acid PC groups. Wrinkle scores in the LLRF group were significantly reduced (by approximately 4 scores) with no scabs being observed, compared with those of the model group. The LRF, L and PC groups showed no statistically significant differences compared to the model group. This indicated that, the combination of LRF and L techniques had exerted a synergistic effect, reducing wrinkles in UVB irradiated mice and restoring them to normal levels seen in untreated mice. Thus, the LLRF treatment provided the best skin treatment, comparable to that of vitamin A acid.

### 3.5 Histological analysis

**Fig. 5:**
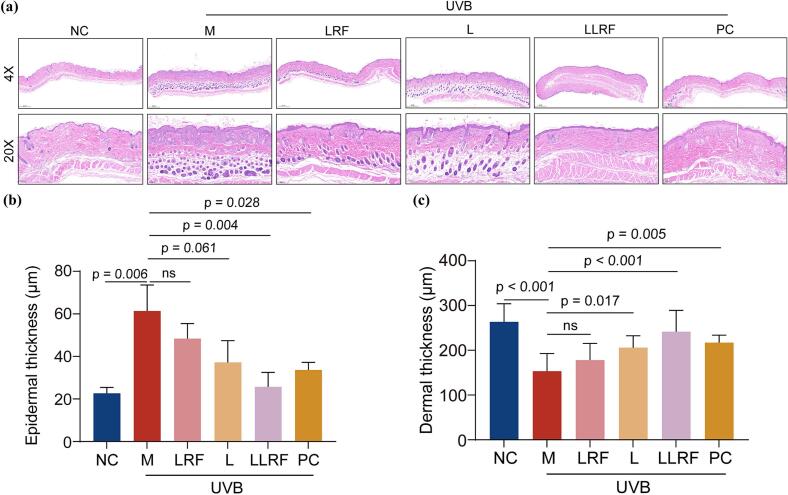
The HE staining of skin for histopathological observation (a) and the thickness of epidermal (b) and dermal (c).

In this study, histopathological changes in the skin of hairless mice were evaluated via HE staining, following which epidermal and dermal thicknesses were measured (Fig. 5). The results shows the epidermal layer of mice in the NC group having a complete skin structure with a normal relatively lesser thickness, and a dermis with wavy collagen fiber which were orderly arranged, evenly distributed, and densely packed, with moderate cell composition. However, the epidermis of mice in the model group showed severe irregular thickening (Qi and Mitragotri, 2019), damage, hyperkeratosis, reduced dermal collagen fiber, degenerate and disorderly arrangement, thickened, curled, broken and unevenly distributed collagen bundles, while the dermis showed a significantly changed composition. Following LRF treatment, the epidermal layer becomes relatively continuous and intact, and excessive keratinization disappeared, but the collagen bundles became thicker and the unevenness of density worsened compared with that of the M group, while the composition of the dermis was also changed moderately. The collagen fibers in the L group were relatively slim and dense, and keratinization was decreased, but the composition in the dermis showed a mild change towards recovery similar to that seen in the LRF group. Following treatment with LLRF, hyperkeratosis disappeared and was replaced with a complete SC, leading to an epidermis with a relatively lesser thickness, slim fibers showing a homogeneous, dense and orderly arrangement, and a dermis with a cell composition which was similar to that of the NC group. In the PC group, keratinization of the epidermis was prevented, and its cell composition recovered, but the collagen bundles in the dermis were relatively rough, unevenly arranged, with inconsistent density. Epidermal thicknesses of the LLRF and PC groups were significantly reduced, compared with that of the model group, and a significant difference was observed between the LRF group or the L group and the model group. The dermal thicknesses of the L, LLRF and PC groups were significantly increased compared with that of the model group, where the LLRF group exhibited the changes leading to the largest increase in thickness. However, dermal thickness changes in the LRF group did not show a significant difference compared to those in the M group. Thus, the combined treatment appears to have recovered dermal thickness following UVB irradiation at a higher level and reduced epidermal thickening. These findings suggest that combination therapy, LLRF, had exerted a significant synergistic effect. Radio-frequency thermal effect offered by LLRF promotes peptide penetration meanwhile, has no adverse effect to SC. This was a non-invasive permenation enhancement method. And it is probably due to the activating of the intracellular signaling pathway leading to dissociation of gap junction and open intercellular space apertures in skin.

**Fig. 6:**
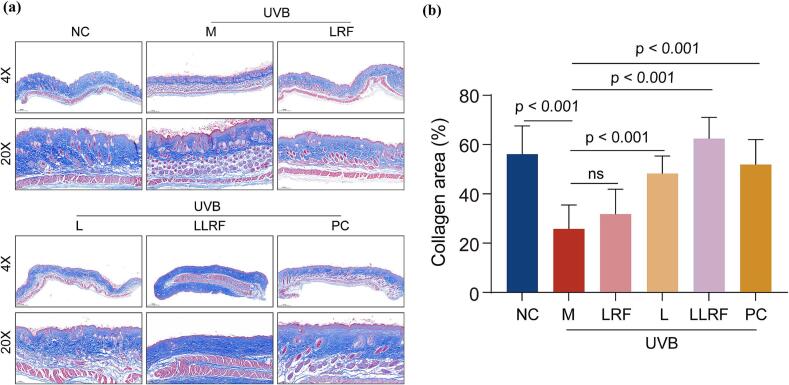
The Masson staining observation(a) and quantitative analysis(b) for skin collagen fibers.

The relative collagen fiber proportions were measured via Masson staining (Fig. 6). The proportion of collagen fibers in the model group was significantly reduced compared with that in the control group, whereas the proportions of collagen fibers in the L, LLRF and PC groups were significantly increased compared with that in the model group, wherein, the increase in amplitude was the largest in the LLRF group, increasing even beyond the proportion in the NC group, indicating that the LLRF treatment exerts a remarkable effect on the formation of collagen fibers. Therefore, the combined treatment appears to exert a significant synergistic antiaging effect in increasing the proportion of collagen fibers in UVB irradiated mice, leading to the recovery of these mice after treatment. Notably, LRF treatment of nude mice exerted only a few promoting effects on collagen fiber formation, due to the low energy/frequency that we adopted, compared to high energy which accelerates the formation of collagen fibers more effectively. Thus, LRF may not have played the leading role in LLRF therapy, the key underlying role being played by the synergistic effect instead. Ultraviolet irradiation affects collagen fibers in two ways: On the one hand, it inhibits collagen synthesis and reduces the content of collagen in the dermis; while on the other hand, it promotes the formation of extracellular matrix degrading enzymes (mainly matrix metalloproteinases such as MMP1 and MMP3), thereby inducing the degradation of collagen, which facilitates the fracturing and disarrangement of collagen fibers (Moon et al., 2000). MMP1 can split type I and type III collagen, and MMP3 further promotes the degradation of collagen (Quan et al., 2009). Therefore, changes in matrix metalloproteinases may provide proof for the synergistic effects exerted by the combined LRF and L therapy method. UVB causes poor keratosis of the epidermis, thickens the spine cell layer and increases epidermal cells, causing the epidermal thickness of the UVB model to increase. This proves that collagen promotes a UVB-induced decline in epidermal barrier function and elasticity. The significant elevation of collagen observed in our study is consistent with the results of stratum corneum barrier function recovery (Fisher et al., 2002).

### 3.6 β-Gal Immunofluorescence detection

**Fig. 7:**
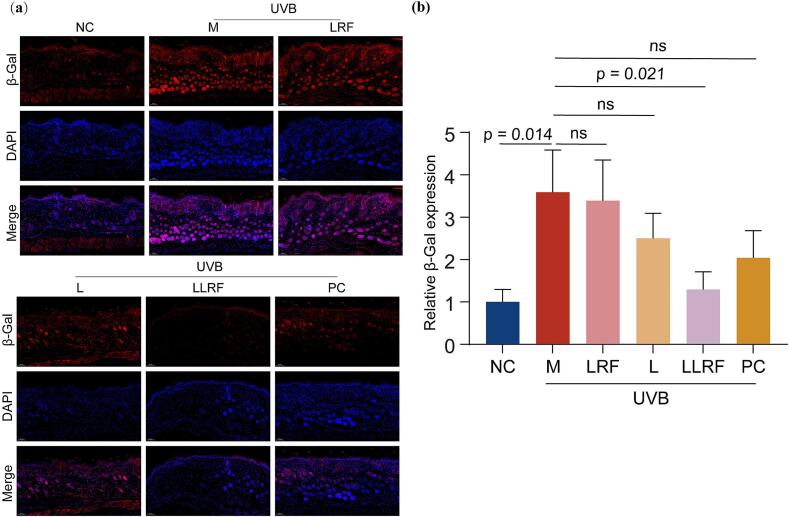
The immunofluorescence observation (a) of β-Gal and its relative expression (b) for different therapy methods.

The immunofluorescence method was adopted to detect the amount of β-Gal expression in mouse skin. The relative expression of β-Gal content in the model group increased significantly, but decreased significantly only in the LLRF group (Fig. 7). There was no significant difference between the LRF, L, or the PC group, and the M group. β-Gal in the combination treatment group returned to the normal levels equivalent to that shown by the NC group. Thus, the LLRF combination therapy appears to have reduced the skin aging marker (β-Gal) of UVB irradiated mice, indicating that. The combination therapy had exerted a significant synergistic effect.

### 3.7 Skin barrier function examination

**Fig. 8:**
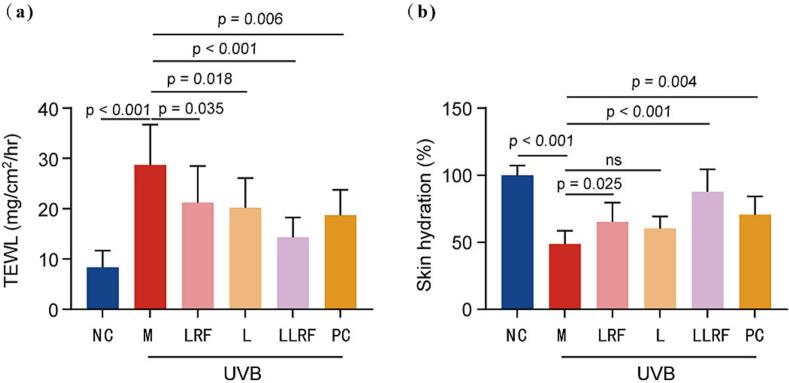
The transepidermal water loss (a) and skin hydration value (b) in hairless mice skin determined for different therapy methods.

The in vivo levels of TEWL and SCH, parameters representative of the mouse skin barrier, were measured using a professional Cutometer, MPA-580 (Fig. 8). The TEWL values of the LRF, L, PC and LLRF groups were all significantly reduced compared with that of the M group, among which the LLRF group exhibited the most prominent reduction in amplitude, resulting in an improvement of approximately 50 %. The hydration measurements of LRF, PC and LLRF groups were all significantly improved, where that of the LLRF group increased by approximately 40 %, with no significant difference being seen between the L and M groups. The downregulation of TEWL value and upregulation of SCH were consistent with previous HE staining-based observations. Combined treatment repairs skin barrier effectively, leading to a reduction in percutaneous water loss following UVB irradiation, and an increase in skin hydration. Accordingly, this indicated that 4 weeks of LLRF treatment, which combined LRF and L technology, had exerted a synergistic effect leading to skin barrier recovery ((TEWL, SCH) in UVB photoaged nude mice.

### 3.8 Real-time PCR

**Fig. 9:**
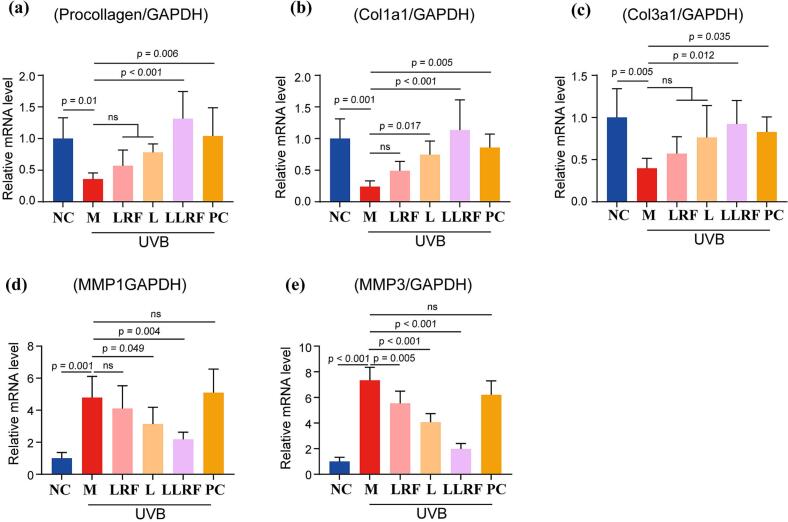
The relative mRNA expression of Procollagen (a), Col1α1 (b), Col3α1 (c), MMP1 (d)and MMP3 (e) corresponding to different therapy methods.

Real-time PCR analysis was performed to clarify mechanisms underlying the synergistic effect of LLRF at the molecular level. The quantitative results are shown (Fig. 9). The relative mRNA expression of procollagen, Col1α1 and Col3α1 in the UVB photoaged model group were all decreased, while the relative mRNA expression of MMP1 and MMP3 were increased, compared to the NC group. The LRF, L, LLRF, and PC groups demonstrated different degrees of improvement compared with the model group, in which, LRF exhibited a poor improvement effect on relevant collagen mRNA expression, but showed a powerful promoting effect on matrix metalloproteinase mRNA expression. On the other hand, the L, PC, and LLRF groups respectively exerted mild, the second strongest and the strongest promoting effects on the expression of genes encoding of three types of collagen and inhibition effects on two type of matrix metalloproteinases, except for that in the PC group, which has no significant effects on mRNA expression of matrix metalloproteinases, even promote it. This may be attributed to the irritation-related side effects of vitamin A acid.

The low-efficiency of the LRF method combined with the mild-efficiency of the L method, led to the highly-efficiency LLRF method, which eliminated disadvantages, such as insufficient inhibition of the expression of matrix metalloproteinases (MMP1 and MMP3), and promoted the expression of collagen encoding genes, thereby exerting a better antiaging effect on mice skin.

### 3.9 Western Blot (WB) test

**Fig. 10:**
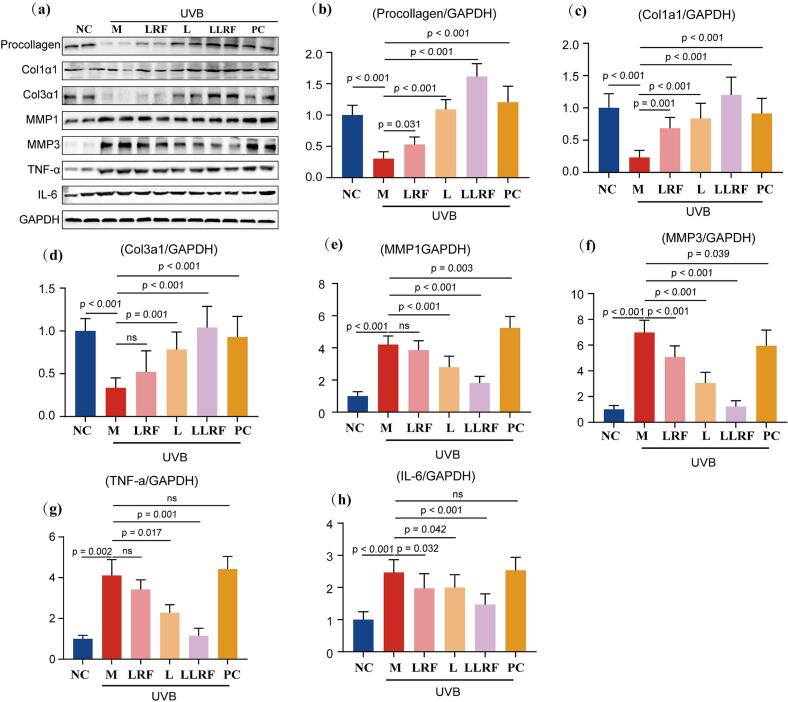
in vitro analysis of the expression levels of different proteins in hairless mice (a), and the relative protein expression of Procollagen (b), Col1α1 (c), Col3α1(d), MMP1 (e), MMP3 (f), TNF-α (g) and IL-6 (h) corresponding to different therapy methods.

Furthermore, relative protein expression was investigated via WB analysis. The results of the relative protein expression analysis showed that the UVB irritation group had undergone massive damage to collagen whether it be Procollagen, Col1α1 or Col3α1,while exhibiting a sharp accumulation of matrix metalloproteinases (MMP1, MMP3) and inflammatory proteins (TNF-α, IL-6) compared with those of the NC group (Fig. 10). By contrast, after 4 weeks of therapy, the LRF, L, LLRF and PC groups, showed varying degrees of improvement against adverse reactions caused by UVB irritation, as shown by an increase in the relative expression of collagen, especially of procollagen and Col1α1. Among them, the LLRF group, the performance of which exceeded that of the NC group, was the most prominent. The relative protein expression of matrix metalloproteinases was inhibited in the LRF (MMP1 not significant), L and LLRF groups, the strongest inhibition effect being exerted by the LLRF group. The protein expression of matrix metalloproteinases did not show any decrease due to vitamin A acid therapy, even exhibiting a moderate increase in MMP1 expression. The relative protein expression of inflammation-related factors (TNF-α, IL-6) revealed results similar to that of the matrix metalloproteinases, with the LLRF group exhibiting the greatest inhibitory effect on TNF-α and IL-6, the effect on TNF-α being more significant than that in IL-6, whereas the PC group did not exert an obvious effect on these two inflammation-related factors, indicating that TNF-α was more closely associated with collagens. Overexpression of both matrix metalloproteinases (MMP1/MMP3) and inflammation-related factors (TNF-α, IL-6) contribute to the degradation of collagens. In this study, the results observed in the PC group revealed that although the expression of matrix metalloproteinases and inflammation-related factors negatively regulate collagens by degrading them, continuous formation of new collagens may balance such degradation. In the above analysis, the anti-aging effect of combined LRF and L treatment showed a “1 + 1 > 2” effect. LLRF promote the penetration of peptide without damaging the SC of the skin, probably related to the activating intracellular signaling pathway followed by the opening of intercellular space apertures, at the same time, there exist synergistic effect between the phisical type of radio-frequency thermal effect caused by LRF and the chemical type of anti-aging effect of peptides in liposome. Thus, combined therapy appears to contribute to protein formation in procollagen, Col1α1 Col3α1, and restore (downregulates) the expression of MMP1 ↓, MMP3 ↓, TNF-α ↓ and IL-6 ↓ in UVB irritated mice compared to those of normal healthy ones.

### 3.10 Clinical trials

**Fig. 11:**
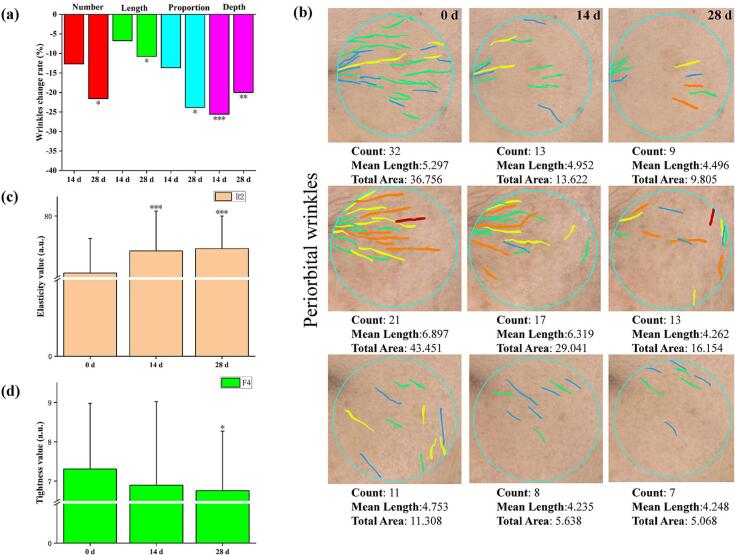
Clinical test results of antiwrinkle (a, b), elasticity (c) and tightness (d) therapy with peptide liposome formulation.

Based on the positive antiaging effects observed in animal experiments, a human clinical trial was conducted to determine the anti-aging ability of L treatment in relation to human facial skin. The wrinkle test for peptide liposome formulation showed a positive result (Fig. 11). The improvement rates of left eye tail stripe length were 6.73 % and 10.77 % following 14 and 28 d of peptide supramolecular liposome extract treatment, respectively, compared with the basic values. The improvement rates of left eye tail striate area were 13.65 % and 23.85 %, following 14 and 28 d of peptide supramolecular liposome extract treatment, respectively, compared with those of the basic values. The improvement rates of the right eye tail stripe depth were 25.56 and 20.00 % following 14 and 28 d, respectively, when the results of the peptide liposome extract was compared with basic values.

The tightness test of peptide loaded liposome formulation also showed a positive result. Compared with the basic values on 14 and 28 d, the elasticity value increased by 8.87 % and 9.80 %, respectively. The improvement rates of the tightness value, F4, were 5.61 % and 7.52 % following peptide liposome extract treatment for 14 and 28 d, respectively, when compared with those of the basic value. The above results sufficiently supported the antiaging effect of peptide liposomes. The clinical trials were approved by Ethics Review Committee of Shenzhen PUJIA Technology Service Co., Ltd. and the permission number is LLSC-2305002.

## 4 Conclusion

The above study may be summarized as follows: A stable peptide liposome with a hydrodynamic diameter of approximately 135 nm and a narrow particle size distribution was prepared. The transdermal penetration experiment indicated that the permeability of LLRF was 3.65 folds higher than that of an unencapsulated peptide, and that the retention of FITC-AH-8 in the skin was significantly increased as shown by fluorescence intensity. FTIR and HE staining to determine the safety of LLRF method in relation to skin histopathology indicated that it was a safer noninvasive transdermal delivery method, and this probably was a diffrent mechanism of open intercellular space apertures in skin and quickly recover, compared with traditional permeation enhancers has a solubilty effect on lipids of SC. In vivo experiments based on mouse UVB models, which monitored multiple indicators, confirmed the significant anti-aging effect exerted by combined therapy involving the LRF technique. A clinical test indicated that the peptide liposome formulation shows significant anti-wrinkle and tightening efficacy.

Our findings also revealed that the PC (vitamin A acid) group promoted collagen production, but exerted only a few inflammation-related factor associated effects or side effects on the skin. LRF treatment alone exerted only a minor effect on the formation of collagen, and did not cause a significant reduction in inflammation-related factors or matrix metalloproteinases. The L (peptide encapsulated liposomes) treatment moderately promoted collagen formation and reduced the production of inflammatory factors and matrix metalloproteinases, but did not exert a significant effect on the expression of collagen III. Notably, the LLRF (low frequency/energy radiofrequency and peptide liposome combination) exerted a powerful and effective synergistic effect which was highly efficient, comprehensive and was safe to use. This synergistic effect was generated by the combining of LRF and L (nano-liposomes carrying antiaging peptides or other substances) technologies.

## Ethics approval and consent to participate

This work received approval from the Ethics Review Committee of Shenzhen PUJIA Technology Service Co., Ltd. (approval number: LLSC-2305002).

## Consent for publication

All authors agree to be published.

## Availability of date and materials

The datasets used or analyzed during the current study are available from the corresponding author on reasonable request.

## Funding

This work was financially supported by the 10.13039/501100001809National Natural Science Foundation of China (Grant Nos. 21905069, 22208073，U21A20307), the Shenzhen Science and Technology Innovation Committee (Grant Nos. ZDSYS20190902093220279, KQTD20170809110344233, GXWD20201230155427003–20200821181245001,GXWD20201230155427003–20200821181809001, ZX20200151), the 10.13039/501100007162Department of Science and Technology of Guangdong Province (Grant No. 2020A1515110879).

## Author's contributions

Nanxi Xiang and Zeting Huang contributed equally to this work. Nanxi Xiang and Zeting Huang designed and jointly completed the experiment and wrote the manuscript. Chunqiao Zhang and Jiahong Huang performed in vivo experiment parts. Zhenyuan Wan and Jichuan Zhang analyzed the data and finished graphical abstract. Chengyu Wu, Jiaheng Zhang and Weihua Peng supervised the study. All authors reviewed the manuscript.

## CRediT authorship contribution statement

**Nanxi Xiang:** Writing – original draft, Project administration, Investigation, Conceptualization. **Zeting Huang:** Writing – original draft, Methodology, Investigation. **Chunqiao Zhang:** Writing – original draft, Investigation. **Zhenyuan Wang:** Methodology. **Jichuan Zhang:** Methodology, Formal analysis. **Chengyu Wu:** Writing – review & editing, Supervision, Project administration, Funding acquisition, Conceptualization. **Weihua Peng:** Writing – review & editing, Supervision, Project administration. **Jiaheng Zhang:** Writing – review & editing, Project administration, Funding acquisition.

## Declaration of competing interest

The authors declare that they have no known competing financial interests or personal relationships that could have appeared to influence the work reported in this paper.

## Data Availability

Data will be made available on request.
